# Two Different Meningioma Variants in the Same Tumor: A Rare Histopathological Finding

**DOI:** 10.1155/2019/8267163

**Published:** 2019-05-09

**Authors:** Reham Khubrani, Sadeq Al-Dandan

**Affiliations:** ^1^Department of Anatomic Pathology, King Faisal Specialist Hospital and Research Center, Riyadh, Saudi Arabia; ^2^Department of Anatomic Pathology, King Fahad Medical City, Riyadh, Saudi Arabia

## Abstract

We report a case of meningioma with both secretory and lipomatous features in an advanced middle aged female patient with a 3-month history of headaches and convulsions. Radiological findings are revised and compared to other reported cases in the literature.

## 1. Introduction

Meningioma is a central nervous system tumor arising from the arachnoid cells present in the arachnoid mater. It is the most common primary tumor of the brain; 33.8% of all primary central nervous system tumors are reported in the United States between 2002 and 2006 [1]. Secretory meningioma (SM) is a rare subtype of meningioma characterized by PAS-positive inclusions [2–4]. While lipomatous meningioma is a rare mesenchymal differentiation among metaplastic meningiomas [5], coexistence of both secretory and lipomatous elements in the same meningioma is even rarer and considered unique. To date, this kind of tumor has not been diagnosed at our institution. To our knowledge, only 3 cases of secretory meningioma with lipomatous component are reported in the literature [6–8].

## 2. Case Report

**Figure 1:**
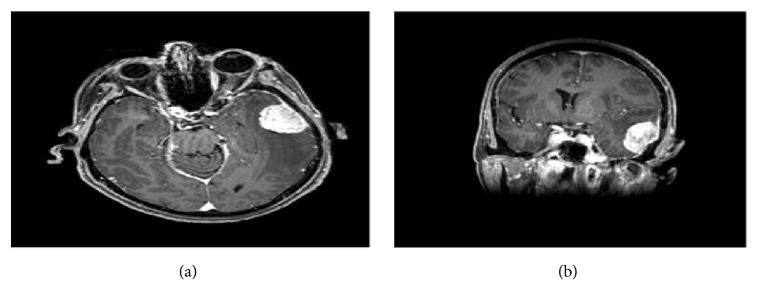
Preoperative magnetic resonant imaging of the brain showing hypertense left-temporal lesion (a) and midline shift and vasogenic edema (b).

A 65-year-old female patient was admitted to the hospital complaining of headaches and convulsions over the previous three months. The patient was conscious and alert with stable vital signs. Computer tomography (CT) scan showed a well-defined left-temporal extra-axial lesion of mixed density, predominantly hypodense (fat density), causing calvarian bone hyperostosis with a localized mass effect on the adjacent temporal lobe. Significant vasogenic edema with associated effacement of the left hemispheric sulci, compression of the ipsilateral ventricle, early left uncal herniation, and a rightward midline shift also were noted. MRI showed a dural-based focal mass in the left-temporal lobe exhibiting a mass effect on the adjacent parenchyma and a large area of perifocal edema (delete vasogenic) causing compression of the ipsilateral ventricle and approximately 6mm midline shift towards the right. Evidence of a high fat component (high T1 and T2 mass) showing avid enhancement with a dural tail sign was present (Figures 1(a) and 1(b)). As a result, the patient underwent a craniotomy and a biopsy was taken.

## 3. Pathological Findings

**Figure 2:**
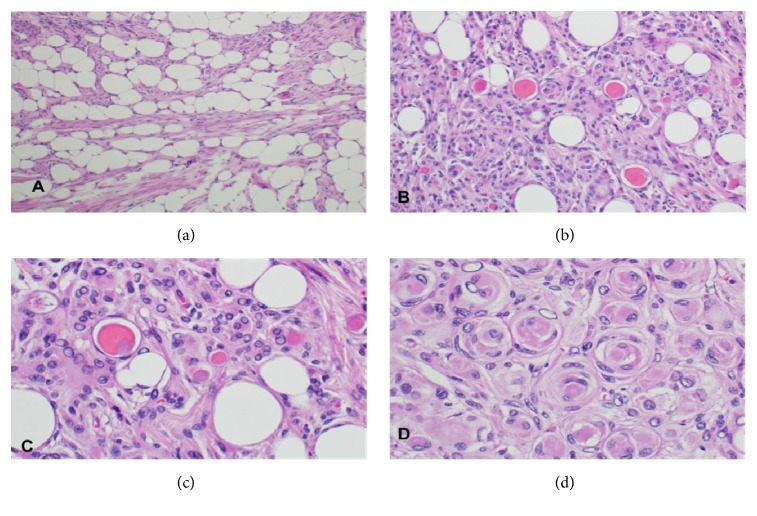
Hematoxylin and eosin stain of the tumor tissue. (a) x100 magnification showing mature adipose tissue intermingling with meningotheliomatous components. (b) x200 magnification demonstrates meningotheliomatous secretory and adipose components. X400 power view of the hyaline inclusion bodies (c) and whorls of the classic meningioma (d).

**Figure 3:**
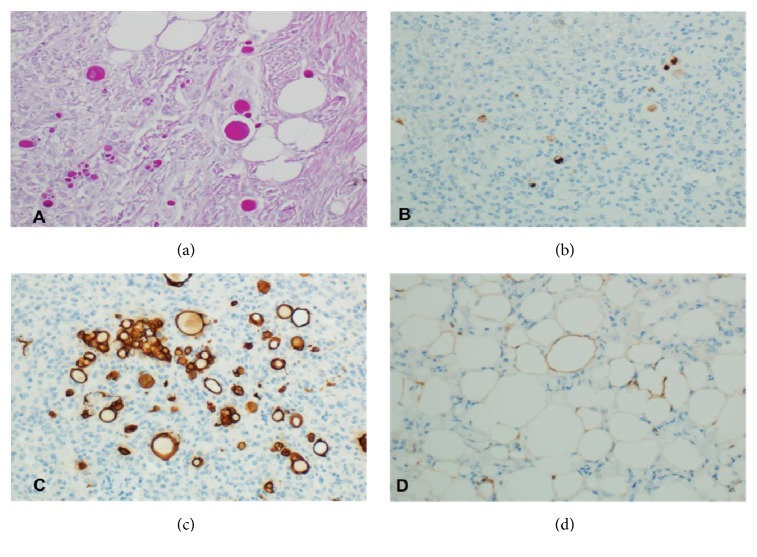
The secretory hyaline bodies highlighted by PAS (a) and CEA (b). Positive reaction of cytokeratin antibody of the cells surrounding the hyaline bodies (c). Mature fat cells positive for S100 (d).

Upon examination in our laboratory, we found a firm, well-circumscribed, white/tan mass attached to the dura. The cut surface showed yellowish fatty tissue interwoven with firm fibrous tissue. Microscopically, the tumor was composed of meningotheliomatous tumor cells with classic whorls in addition to hyaline inclusion bodies scattered throughout the examined tissue, representing the secretory component (Figures 2(b) and 2(c)). These hyaline bodies were positive for periodic acid Schiff (PAS) stain (Figure 3(a)) as well as carcinoembryonic antigen (CEA) immunohistochemical stain (Figure 3(b)). The tumor cells surrounding these hyaline inclusions tested positive for cytokeratin (Figure 3(c)). Another significant component was mature adipose tissue composed of cells with large, clear cytoplasmic droplets, and these cells were positive for S100 (Figure 3(d)).

## 4. Discussion

Although meningothelial meningioma is quite common, the presence of secretory and lipomatous features in the same tumor is exceptional and deserves studying and reporting. Secretory meningioma is identified radiologically by predominantly hypo-to iso-signal T1 weighted and hypersignal T2 weighted (T2 flair) MR images. These findings have been related to soft tissue consistency and the severe peritumoral brain edema. The predominant finding on MR images is a strongly enhanced, homogeneous tumor. Dai-Jun Wang and his colleagues call this enhancement, “xenon light” like enhancement comparable to the enhancement of secretory meningiomas [9]. Histologically, secretory meningioma shows CEA and PAS-positive hyaline inclusion bodies, with surrounding CK-positive tumor cells. [2–4]. Lipomatous meningioma is characterized radiologically by hypodensity on CT scan and bright hyperintensity on both T1- and T2-weighted MRI images, and it is always enhanced [4]. Histologically, this tumor consists of adipocyte-like cells within conventional (meningothelial) meningioma. This type of cell may be found in meningothelial and transitional variants, although it is unusual to find such cells accompanying secretory variant [4]. Whether these adipocyte-like cells result from an advanced lipidization of neoplastic meningothelial cells or a true metaplastic transformation of meningothelial cells into mature fat tissue is an area of debates. There are more agreements for the former, frequently based on immunohistochemical and ultrastructural findings [2, 4, 10].

Our case demonstrates both secretory and lipomatous elements within conventional meningioma, as demonstrated by both radiological and pathological studies. It shows mixed density on CT scan. MRI revealed evidence of both peritumoral brain edema and fat components. The key features of our case in comparison with the previous reported cases are summarized in the table (Table 1). Each of the five cases reports on female patients with a mean age of 58 years, a feature similar to that of conventional meningioma. All tumors arise in the brain hemisphere (2 in the frontal lobe and 2 in the temporal lobe). Headache was the main presenting symptom in three cases, convulsion in two, and loss of consciousness in one. The three previous cases as well as the current case show fat intensity and peritumoral brain edema on the radiological study. Additional reporting and investigation of similar tumor are required.

## Figures and Tables

**Table 1:** Comparison between the current case (case 4) and the previously reported cases.

	Case 1	Case 2	Case 3	Current case (case 4)
age	67	43	58	65

Gender	female	female	female	female

tumor site	right frontal lobe	left frontal lobe	right hemisphere	left temporal lobe

presenting symptom	headache	convulsion and unconsciousness	headache	headache and convulsion

outcome	x	uneventful	hydrocephalus	hydrocephalus
